# Poor recognition of O6-isopropyl dG by MGMT triggers double strand break-mediated cell death and micronucleus induction in *FANC*-deficient cells

**DOI:** 10.18632/oncotarget.10928

**Published:** 2016-07-29

**Authors:** Kiyohiro Hashimoto, Vyom Sharma, Hiroyuki Sasanuma, Xu Tian, Minoru Takata, Shunichi Takeda, James A. Swenberg, Jun Nakamura

**Affiliations:** ^1^ Department of Environmental Sciences and Engineering, University of North Carolina at Chapel Hill, Chapel Hill, NC 27516, USA; ^2^ Drug Safety Research Laboratories, Pharmaceutical Research Division, Takeda Pharmaceutical Company Limited, Fujisawa, Kanagawa 251-8555, Japan; ^3^ Department of Radiation Genetics, Kyoto University, Graduate School of Medicine, Yoshida Konoe, Sakyo-ku, Kyoto 606-8501, Japan; ^4^ Laboratory of DNA Damage Signaling, Department of Late Effects Studies, Radiation Biology Center, Kyoto University, Graduate School of Medicine, Yoshida Konoe, Sakyo-ku, Kyoto 606-8501, Japan

**Keywords:** isopropyl methanesulfonate, alkylation, DT40, FANC, micronucleus

## Abstract

Isopropyl methanesulfonate (IPMS) is the most potent genotoxic compound among methanesulfonic acid esters. The genotoxic potential of alkyl sulfonate esters is believed to be due to their alkylating ability of the O6 position of guanine. Understanding the primary repair pathway activated in response to IPMS-induced DNA damage is important to profile the genotoxic potential of IPMS. In the present study, both chicken DT40 and human TK6 cell-based DNA damage response (DDR) assays revealed that dysfunction of the FANC pathway resulted in higher sensitivity to IPMS compared to EMS or MMS. O6-alkyl dG is primarily repaired by methyl guanine methyltransferase (MGMT), while isopropyl dG is less likely to be a substrate for MGMT. Comparison of the cytotoxic potential of IPMS and its isomer n-propyl methanesulfonate (nPMS) revealed that the isopropyl moiety avoids recognition by MGMT and leads to higher cytotoxicity. Next, the micronucleus (MN) assay showed that *FANC* deficiency increases the sensitivity of DT40 cells to MN induction by IPMS. Pretreatment with O6-benzyl guanine (OBG), an inhibitor of MGMT, increased the MN frequency in DT40 cells treated with nPMS, but not IPMS. Lastly, IPMS induced more double strand breaks in *FANC*-deficient cells compared to wild-type cells in a time-dependent manner. All together, these results suggest that IPMS-derived O6-isopropyl dG escapes recognition by MGMT, and the unrepaired DNA damage leads to double strand breaks, resulting in MN induction. FANC, therefore, plays a pivotal role in preventing MN induction and cell death caused by IPMS.

## INTRODUCTION

Isopropyl methane sulfonate (IPMS), an alkyl sulfonate, is a potential genotoxic impurity (GTI) that can form as a byproduct during the synthesis of sulfonate salts when sulfonic acids react with isopropanol, a low molecular weight alcohol [1]. IPMS displays genotoxicity and carcinogenicity in *in vitro* and *in vivo* assays, and it is categorized as the most potent mutagen in the Ames and *in vivo* micronucleus assays [2–9]. Despite its hazardous profile, there has been little attention on IPMS compared to what is known about methyl methanesulfonate (MMS) and ethyl methanesulfonate (EMS), which are also potential GTIs. These alkyl sulfonates constitute a representative class of direct mutagens whose genotoxicity is attributed to their alkylating ability at the O6 position of dG [5, 10]. The genotoxicity of IPMS has been hypothesized to be attributed to the differences in the S_N_1/S_N_2 reaction type and the Swain Scott constants [11], as compared to MMS and EMS [5]. Although IPMS-mediated DNA adduct formation has been previously studied, it is important to determine its net biological effect (*e.g.* cytotoxicity and genotoxicity outcome), which is determined by the balance between the generation of DNA damage and the DNA repair efficiency. Understanding both the damage and repair aspects helps to more accurately interpret how individual alkylating agents induce genotoxicity. In this study, we conducted the DNA damage response (DDR) assay using isogenic chicken DT40 cell lines [12–14] to understand the repair or tolerant pathway activated in response to IPMS. DT40 cells originated from a chicken B-lymphocyte line derived from an avian leucosis virus-induced bursal lymphoma isolated in 1985 [15]. The isogenic DT40 cell lines in this study broadly probe biological targets, pathways and mechanisms in relation to genotoxicity and/or cytotoxicity endpoints for a large number of chemicals [16, 17]. The DDR assay, which examines cytotoxicity in DNA repair-deficient DT40 mutants *versus* the parental DT40 cells, is a rapid and simple method to evaluate the genotoxicity of xenobiotics.

Interestingly, small differences in chemical structure can drastically change genotoxicity. nPMS is an isomer of IPMS with a straight chain in the alkyl side chain structure, while IPMS has an isopropyl moiety. Despite the subtle change in structure, the genotoxic potential of nPMS is significantly weaker than IPMS [2, 4–6, 8, 9]. The difference in the activities of these two agents has not been adequately explained, but it is believed to be due to a combination of the DNA lesion-forming potential and repair or tolerance capability. A possible explanation for the different efficiencies in the formation of DNA adducts is that IPMS is able to form a carbonium ion (S_N_1) while the reactivity of nPMS occurs *via* a bimolecular nucleophilic displacement reaction (S_N_2). The S_N_1 reactivity of IPMS indicates that it possesses stronger reactivity at the O6 position of dG compared to nPMS [18]. As a result, IPMS is believed to generate more DNA adducts at the O6 position of dG than nPMS. Thus, the S_N_1/S_N_2 reaction type and the Swain Scott constants are useful values for predicting the potential for genotoxicity. However, as previously mentioned, genotoxicity is characterized not only by the generation of DNA damage but also the effect on DNA damage repair; therefore, it is important to characterize the changes in repair or tolerance capabilities after IPMS exposure, which have not been previously highlighted.

Alkylating agents predominantly form adducts at N- and O- atoms, and O-alkylations (*e.g.*, O6-alkyl dG) are known to be highly mutagenic and clastogenic. Although its proportion is less than 10% of the total number of DNA methyl adducts, O-alkylations are stable and persist in the absence of the DNA repair protein O6-methylguanine-DNA methyltransferase (MGMT) [19, 20]. The MGMT enzyme repairs these adducts by transferring the alkyl group from the oxygen in the guanine to a cysteine residue in the catalytic pocket of MGMT [21]. Exposure to IPMS results in bulkier adducts, which are slower to repair than those induced by ENU [22–24]. Dolan *et al*. reported that MGMT efficiently removes O6-n-propyl guanine, but its activity on O6-isopropyl dG is less extensive [25]. It is not clear how differences in MGMT repair efficiency impacts the cellular response. To address this point, we inhibited MGMT activity in cells, exposed these cells to IPMS, nPMS or MMS, and examined the cytotoxic or genotoxic activity. Because O6-n-propyl dG and O6-methyl dG are more effectively repaired by MGMT than O6-isopropyl dG, MGMT depletion would be expected to generate different responses to each chemical. In this study, cells were pre-incubated with O6-benzylguanine (OBG), a MGMT inhibitor, and the LC_50_ and MN induction in DT40 cells treated with IPMS, nPMS or MMS were measured as indices of cytotoxicity and genotoxicity.

Unrepaired O6-alkyl dG forms mispairings with deoxythymidine and is recognized by mismatch repair (MMR); however, MMR cannot repair O6-methylG/T mispairs, which eventually lead to double-strand breaks (DSBs). Because O6-isopropyl dG is poorly recognized by MGMT, it is highly likely to form mispairings and form DSBs. It has been reported that DSB repair pathways are activated by methylating agents [26]. Taking this into consideration, we measured γ-H2AX as an indicator for formation of DSBs. Wild-type and sensitive mutant cells were exposed to IPMS, and the population of γ-H2AX positive cells was measured to investigate whether the DSBs induced by IPMS causes the unique response found in the DDR assay.

## RESULTS

### Greater cytotoxicity of IPMS in *FANC*-deficient DT40 cells compared to MMS or EMS

**Figure 1:**
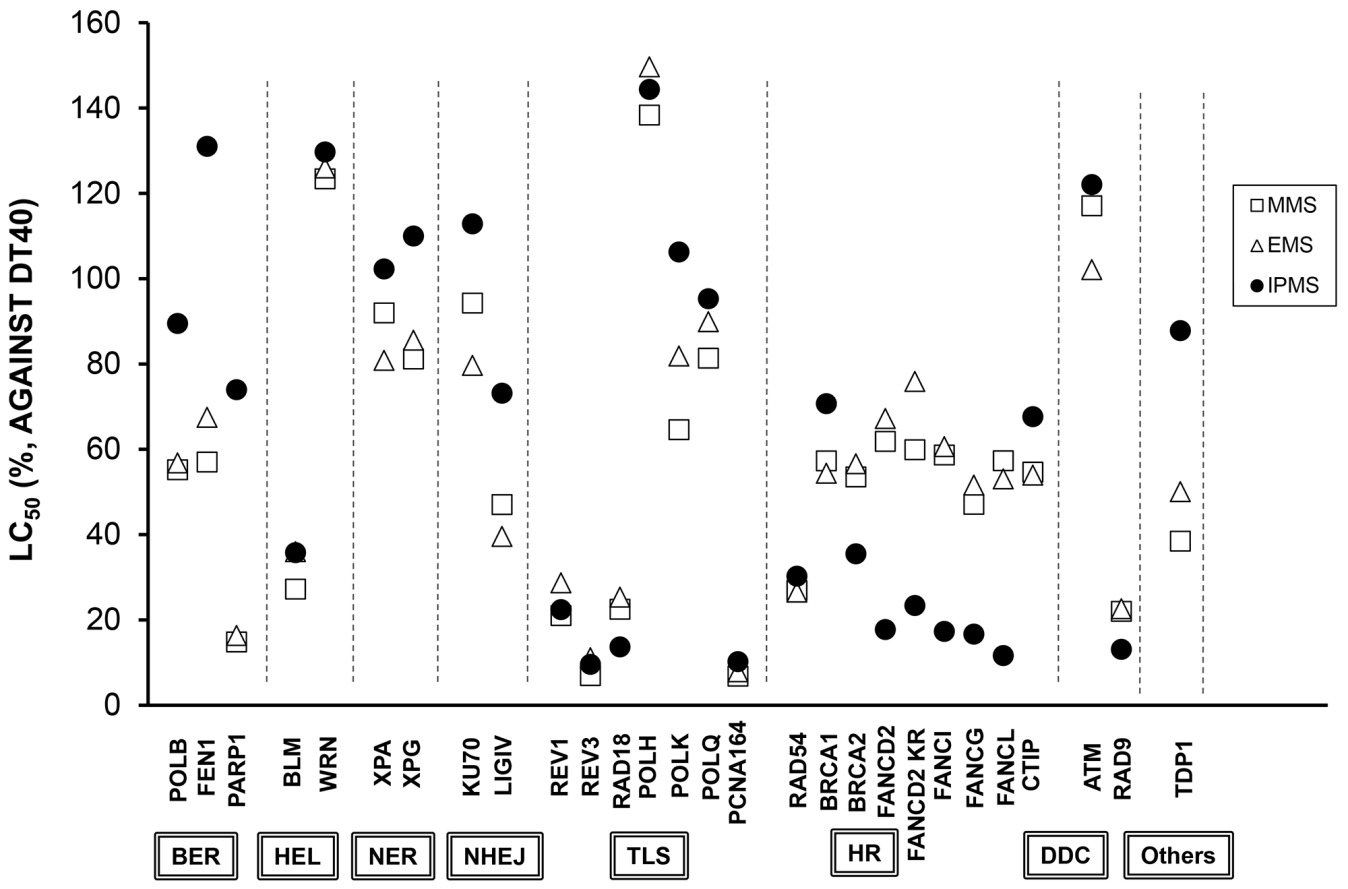
DT40 cell-based DNA damage response assay for IPMS, MMS and EMS Each mutant cell line lacking a specific gene was exposed to serial dilutions of each chemical, and the relative LC_50_ was normalized based on the LC_50_ in DT40 wild-type cells. The data shows the mean of at least three independent experiments. See Table 1 for the abbreviation of the DNA repair or tolerance pathways.

To determine the repair or tolerance mechanism of IPMS-induce DNA damage, twenty-eight isogenic mutant cells were exposed to IPMS. The mutant cell lines cover a variety of DNA repair/tolerance pathways (*e.g.,* BER, base excision repair; HEL, helicase; NER, nucleotide excision repair; NHEJ, non-homologous end-joining; TLS, translesion DNA synthesis; HR, homologous recombination; DDC, DNA damage checkpoint). Considering the weaker S_N_1-reactivity and stronger S_N_2-reactivity of MMS and EMS, we also exposed cells to these chemicals in order to see if the S_N_-1 dominant IPMS gave a different response from MMS and EMS. The LC_50_ in each mutant was compared to that in DT40 cells. The mean LC_50_'s in parental DT40 cells were 736 μM, 65.7 μM, and 1234 μM for IPMS, MMS and EMS, respectively. For the mutant cell lines, the average of 3 or more independent experiments are plotted as the relative LC_50_ (Figure 1). Among these cell lines, DT40 cells deficient in *FANC* genes showed greater sensitivity to IPMS than to MMS or EMS. The relative LC_50_ of MMS or EMS in *FANC*-deficient cells was approximately 60%, while the value dropped to a range of 10-40% in *FANC*-deficient cells treated with IPMS. On the other hand, IPMS showed moderate to no toxicity in the *POLβ-*, *FEN1-*, and *PARP1*-deficient DT40 cells, while MMS or EMS showed obvious toxicity in these cells, suggesting that the BER pathways are less involved in the repair of IPMS-induced DNA damage. For the other mutant cells, there was no difference in the relative LC_50_ among all of the three sulfonic acids tested. Interestingly, the LC_50_ values in MMS- and EMS-treated mutants were comparable, suggesting that the DDR to these two chemicals are quite similar.

**Table 1:** A list of examined DT40 mutant cell lines

DNA repair/tolerance pathway	Genes
Base Excision Repair (BER)	*POLβ, FEN1, PARP1*
RecQ like Helicase	*BLM, WRN*
Nucleotide Excision Repair (NER)	*XPA, XPG*
Non-Homologous End-Joining (NHEJ)	*LIGIV, KU70*
Homologous Recombination (HR)	*RAD54, BRCA1, BRCA2, FANCI, FANCG, FANCC, FANCL, FANCD2, FANCD2-K563R* (monoubiquitination-deficient mutant), *CTIP*
DNA damage checkpoint (DDC)	*RAD9, ATM*
Trans-Lesion Synthesis (TLS)	*REV1, REV3, POLH, POLK, POLQ, RAD18, PCNA K164R* (monoubiquitination-deficient mutant)
Other	*TDP1*

### The involvement of the *FANC* pathway in the repair and tolerance of IPMS-induced DNA damage

**Figure 2:**
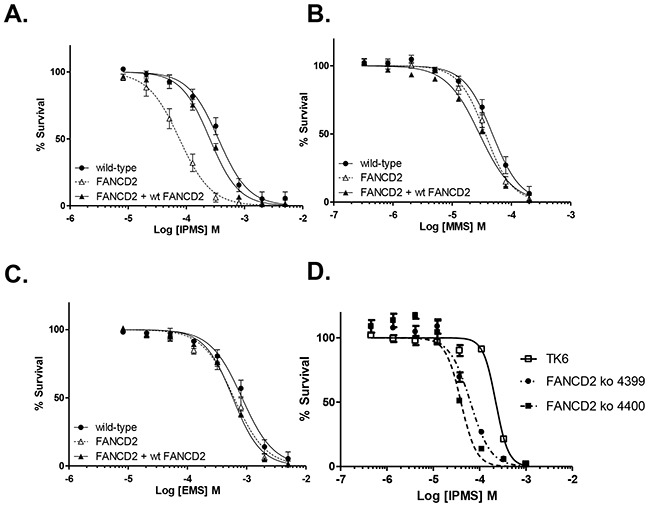
FANCD2 plays a critical role on cell survival in the presence of IPMS *FANCD2*-deficient cells transfected with the *FANCD2* plasmid were exposed to IPMS **A.** MMS **B.** or EMS **C.** at 8 concentrations in parallel with *FANCD2* knock-out and wild-type cells. **D.** *FANCD2* knock-out (two clones) and *wild-type* TK6 cells were treated with IPMS. After treatment, cell viability was evaluated with XTT, and the relative survival at each concentration was calculated. The data shows the mean and standard deviation of at least three independent experiments.

In order to confirm that the hypersensitivity of DT40 cells to IPMS results from a dysfunction in the FANC pathway, *FANCD2*-deficient cells were transfected with a functional *FANCD2* plasmid and treated with IPMS, MMS or EMS. The sensitivity of these *FANC2*-deficient cells was compared with wild-type cells to determine if ectopic expression of *FANC2* rescues its DNA damage repair function. After IPMS treatment, the survival curve of *FANCD2*-deficient cells transfected with the *FANCD2* plasmid is similar to that of wild-type cells and is significantly different from the survival curve of *FANCD2* mutant cells (Figure 2A). The obtained LC_50_ values were 75.99 μM, 253.4 μM and 355.1 μM in *FANCD2*, *FANCD2*+wt *FANCD2* and wild-type cells, respectively. These results indicate that ectopic expression of *FANCD2* restored the resistance to IPMS. In contrast, no obvious difference was observed on the survival curves between *FANCD2* and *FANCD2*+wt *FANCD2* cells treated with MMS and EMS (Figure 2B and 2C). IPMS also caused hyper-sensitivity to cells expressing monoubiquitination-deficient FANCD2 (FANCD2 K563R) (Figure 1). Thus, these results strengthen our findings that the FANCD2, or more specifically monoubiquitination of FANCD2 at lysine 563, is critical for tolerating IPMS-induced DNA damage. We then addressed whether human cells deficient in FANCD2 are also hyper-sensitive to IPMS. To test this question, *FANCD2* gene was deleted in human TK6 lymphoblastoid cells (Supplementary Figure S1). As with the DT40 cells, TK6 *FANCD2* knock-out cells were markedly sensitive to IPMS in terms of cell toxicity (Figure 2D). Together, these results suggest that the FANC pathway plays a pivotal role in the repair or tolerance of IPMS-induced DNA damage.

### The isopropyl moiety plays a crucial role in DNA damage response to IPMS in the absence of FANC repair machinery

**Figure 3:**
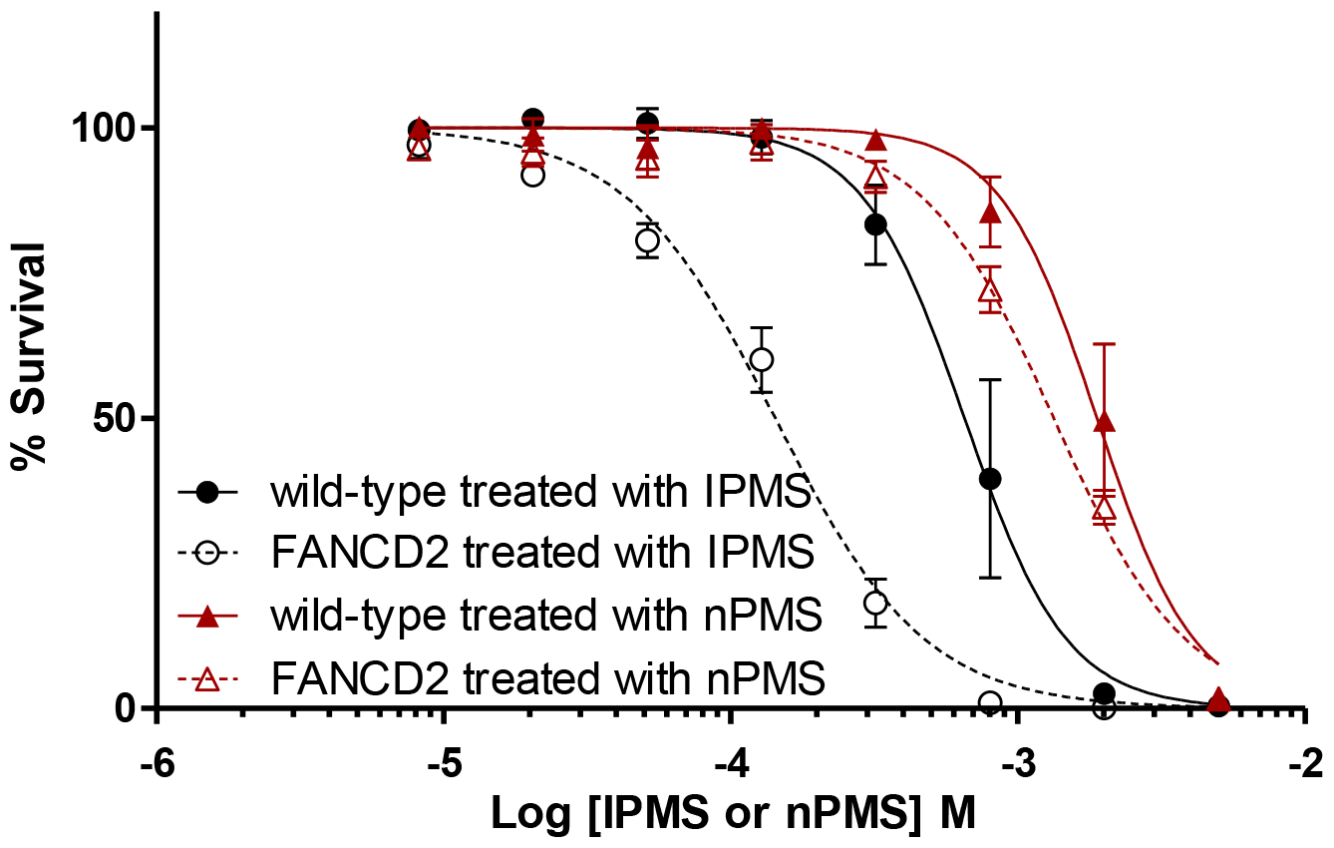
Differential toxicity of IPMS and nPMS in *FANCD2*-deficient DT40 cells The cytotoxicity of IPMS and nPMS in *FANCD2*-deficient cells was examined. *FANCD2*-deficient cells and wild-type cells were exposed to IPMS and nPMS at 8 concentrations. After treatment, cell viability was evaluated with XTT, and the relative survival at each concentration was calculated. The data shows the mean and standard deviation of at least three independent experiments.

To understand how the subtle change in chemical structure between IPMS and nPMS gives different cellular responses, we examined the sensitivity of *FANC*-deficient cells to nPMS, which possesses the n-propyl moiety instead of the isopropyl moiety present in IPMS. The role of the FANC repair pathway in nPMS-derived DNA damage was investigated in comparison with IPMS-derived DNA damage. The survival curve of *FANCD2* cells treated with IPMS is significantly different from that of wild-type cells while this difference was not observed when cells were treated with nPMS (Figure 3). The LC_50_ values were 1879 μM and 1421 μM in wild-type and *FANCD2* cells treated with nPMS, whereas the values were 706 μM and 142 μM in wild-type and *FANCD2* cells treated with IPMS. No obvious difference in sensitivity to nPMS was observed in *FANCG*, *FANCI*, and *FANCL* compared to that in wild-type cells, suggesting that the FANC repair pathway is not the primary repair or tolerance pathway activated in response to nPMS-induced DNA damage.

### No involvement of the MGMT repair pathway in IPMS-derived DNA damage in DT40 cells

**Figure 4:**
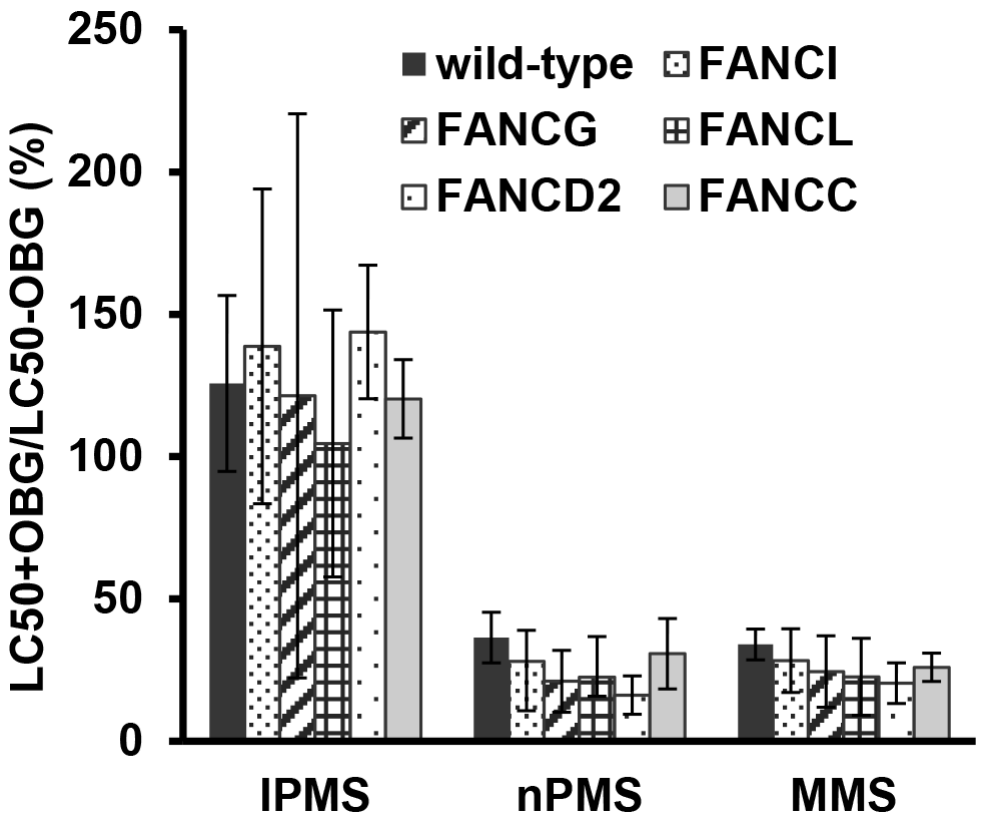
Impact of MGMT function on the toxicity of IPMS, nPMS, and MMS in DT40 cells deficient and proficient in *FANC* genes Wild-type and *FANC*-deficient cells with or without O6-benzylguanine pre-treatment were exposed to IPMS, nPMS or MMS. After treatment, XTT was added, and cell viability was measured. Based on the cell viability data, the LC_50_ was calculated in each treatment condition. The LC_50_ with pre-treatment is divided by the LC_50_ without pre-treatment and expressed as percentage. The data shows the mean and standard deviation of at least three independent experiments.

To investigate the involvement of the MGMT repair pathway on IPMS- and nPMS-derived DNA damage and consequent cell death, DT40 wild-type cells pretreated with O6-benzyl guanine (OBG), which inhibits MGMT activity, were exposed to IPMS and nPMS. To see if inactivation of the MGMT repair pathway further potentiates the cytotoxicity of alkylating agents, the LC_50_ values in the OBG pretreated group were compared with those in the untreated group. As a well-known alkylating agent, MMS was exposed in parallel. To determine if the FANC and MGMT repair pathways work independently, *FANCI-, FANCG-, FANCL-, FANCC-,* and *FANCD2-* deficient cells were also included in the assay. As a result, OBG pretreatment greatly reduced the LC_50_ values in all of the examined cells treated with nPMS and MMS, but not IPMS (Figure 4). These results are consistent with the fact that MGMT recognizes O6-methyl, ethyl, and propyl dG [25]. It is conceivable, therefore, that because O6-isopropyl dG is poorly recognized by MGMT, MGMT depletion does not change the LC_50_, while O6-n-propyl dG or O6-methyl dG, which are recognized by MGMT, greatly reduce the LC_50_ in the pretreated cells. The same pattern was observed in the *FANC*-deficient mutants.

### Greater MN induction in *FANC*-deficient cells treated with IPMS

**Figure 5:**
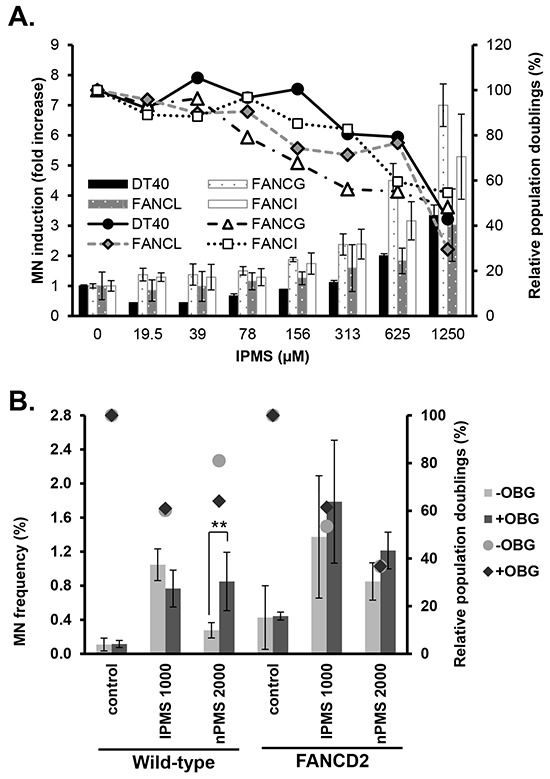
Micronucleus induction in wild-type and *FANC*-deficient cells treated with IPMS **A.** The fold increase in MN frequency over the control in DT40 wild-type, *FANCI, FANCG* and *FANCL* mutants treated with IPMS for 16 hours is shown. The relative population doublings are also plotted. The data shows the mean and standard deviation of two independent experiments. **B.** This experiment was conducted to examine the effect of MGMT inhibition on micronucleus induction in wild-type and *FANCD2*-deficient cells treated with IPMS or nPMS. Wild-type or *FANCD2*-deficient cells with or without O6-benzylguanine pre-treatment were exposed to IPMS or nPMS. After 16 hours of treatment, cells were harvested and stained with fluorescence dye for the flow cytometry-based MN assay. Statistical analyses were conducted for the MN frequency comparing the pre-treated samples against the samples without pretreatment. The relative population doublings are also plotted. The data shows the mean and standard deviation of two independent experiments.

The MN assay with IPMS was conducted in wild-type and *FANC*-deficient cells to determine the impact of a dysfunction in the FANC pathway on MN induction. Results revealed MN induction by IPMS treatment in both wild-type and *FANC*-deficient mutant cells (Figure 5A). The point of departure (PoD) analysis revealed that the break point concentration of MN induction over the baseline level was lower in *FANCG-*, *FANCI-* and *FANCL-*deficient cells compared to that of wild-type cells, with the calculated PoD being 81 μM, 56 μM, and 51 μM in *FANCG*, *FANCI* and *FANCL* cells, respectively, as compared to 110 μM in DT40 wild-type cells. The PoD value indicates the concentration in which a biological effect is observed. Therefore, the lower PoD values observed in *FANC*-deficient cells indicate that the FANC pathway contributes to suppressing MN formation upon IPMS treatment. We also investigated the impact of MGMT inhibition on MN induction by IPMS or nPMS to examine whether the differential recognition between O6-isopropyl dG and O6-n-propyl dG by MGMT influences the MN results, as observed in the cytotoxicity assay. Cells pretreated with OBG were exposed to IPMS or nPMS, and the MN frequency was measured. As a result of two independent experiments, both chemicals increased the MN frequency in both wild-type and *FANCD2*-deficient cells (Figure 5B). The spontaneous MN frequency was higher in *FANCD2* cells (0.4%) than in wild-type cells (0.1%), suggesting that the FANC pathway plays a protective role against spontaneously occurring DNA damage. The OBG pretreatment increased the MN frequency in wild-type cells treated with nPMS, but it did not increase the frequency in wild-type nor *FANCD2*-deficient cells treated with IPMS. The OBG pretreatment also reduced the relative population doubling in wild-type cells treated with nPMS, but not for IPMS-treated cells. In the *FANCD2* cells treated with nPMS, OBG pretreatment slightly increased the MN frequency, but the effect was not significant. These results indicate that, in contrast to O6-isopropyl dG, MTMT removal of O6-n-propyl dG reduces MN formation.

### Induction of DSBs in DT40 and TK6 cells treated with IPMS

**Figure 6:**
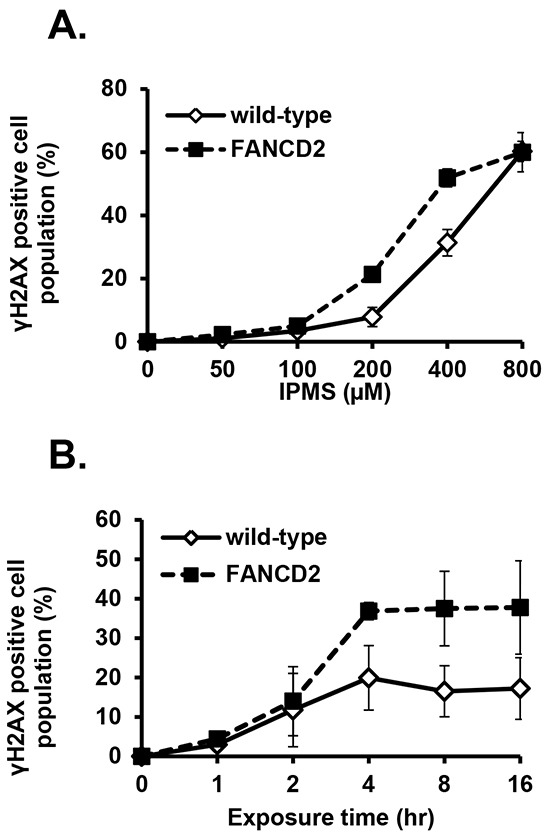
IPMS-induced γH2AX in DT40 cells with or without functional FANCD2 **A.** Dose response of γH2AX was examined in wild-type and *FANCD2*-deficient cells treated with IPMS. Cells were treated with IPMS for 4 hours, and the γH2AX expression was measured. The y-coordinate indicates the population of γH2AX-positive cells. Each measurement represents the mean and standard deviation of two independent experiments. **B.** Expression of γH2AX was examined in a time-dependent manner in wild-type and *FANCD2*-deficient cells treated with IPMS. The kinetics of γH2AX induction was investigated after treatment with 400 μM IPMS in wild-type and *FANCD2*-deficient cells. Cells were exposed to IPMS at different times at 39°C, and cells were harvested thereafter for immunostaining and flow cytometry-based analyses. Each measurement represents the mean of two independent experiments.

**Figure 7:**
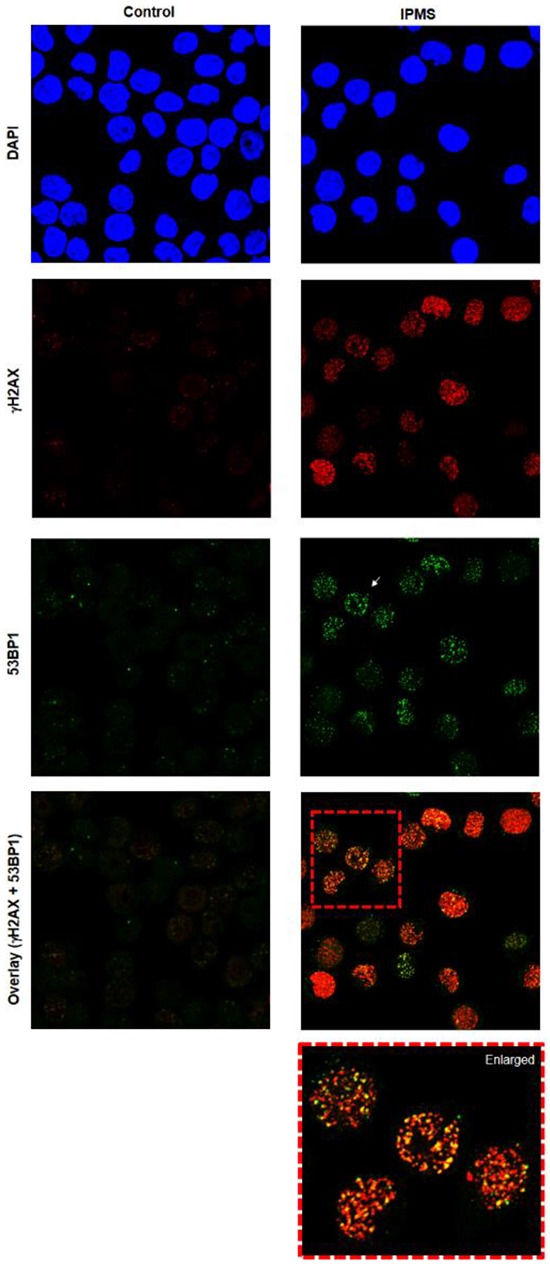
Induction of 53BP1 and γH2AX foci in TK6 cells following exposure to IPMS TK6 cells were exposed to IPMS (750 μM) for 24h and co-immunostained with anti-53BP1 (green) and anti- γH2AX (red). Nuclei were counterstained with DAPI (blue). The images were captured using confocal microscopy and represents maximum intensity projections of z-stacks. Magnification, 600X.

Next, we addressed whether O6-isopropyl dG generates DSBs and if the inability to repair DSBs in FANC-deficient cells results in an accumulation of DSBs, a higher incidence of cell death, and MN induction by IPMS. To do this, we analyzed the population of γH2AX-positive cells in wild-type and *FANCD2*-deficient cells. After a 4-hour treatment with IPMS, the population of γH2AX-positive cells in both wild-type and *FANCD2*-deficient cells showed a linear dose response at a concentration of 100 μM or higher (Figure 6A). In cells that were not treated with IPMS, 4.2 and 11.6% of wild-type and *FANCD2*-deficient cells were positive for γH2AX, respectively. The evidence for FANC involvement in DSB repair [27] is consistent with our observation that *FANCD2*-deficient cells display a higher incidence of γH2AX. To estimate the number of IPMS-induced γH2AX-positive cells, these baseline values were subtracted from the observed values at each time and dose point. Time course experiments revealed that there was an increase in γH2AX-positive cells within 1 hour of treatment in both wild-type and *FANCD2*-deficient cells treated with 400 μM IPMS. The number of γH2AX-positive cells peaked at 4 hours after treatment, and this peak was maintained thereafter. In *FANCD2*-deficient cells, the percentage of γH2AX-positive cells is almost twice that of wild-type cells at the 4- and 6-hour treatment time points (Figure 6B). We also examined nuclear γ-H2AX and 53BP1 foci formation in TK6 cells after 24-hour exposure to IPMS. A marked increase in both γ-H2AX and 53BP1 foci formation was observed in IPMS-exposed cells compared to control (Figure 7). To test if both γ-H2AX and 53BP1 are co-localized as foci, we performed co-immunostaining of TK6 cells with γ-H2AX and 53BP1. Many γ-H2AX foci were co-localized with 53BP1 foci, indicating that IPMS causes DSBs in human TK6 cells as well.

## DISCUSSION

The present study was conducted to elucidate the repair or tolerance pathway activated in response to IPMS and to understand how O6-isopropyl dG leads to cell death and/or genomic damage. To characterize cellular response to IPMS treatment, we compared the genotoxicity and cytotoxity potential between IPMS and nPMS, which is an isomer of IPMS that lacks the isopropyl moiety and exhibits weaker S_N_-1 reactivity and stronger S_N_-2 reactivity. Due to the higher S_N_1-reactivity of IPMS, we hypothesized a greater percentage of O6 adducts from IPMS treatment. Comparison of the sensitivity of wild-type and *FANCD2*-deficient cells to IPMS and nPMS revealed that the branched alkyl moiety of IPMS is a key structure that mediates the hypersensitivity of *FANC*-deficient cells to IPMS, and that the cellular response to nPMS is similar to that of MMS and EMS. Alkyl groups in O6-alkyl dG adducts are repaired by MGMT; however, the repair efficiency of this enzyme varies depending on the substrate. It has previously been seen that O6-isopropyl dG is less extensively repaired than O6-methyl dG or O6-ethyl dG [28], but it remained unclear how this difference in repair efficiency affects cellular responses (*e.g.* measured as cytotoxicity, mutagenicity and clastogenicity). To examine the impact of differences in efficiencies of the MGMT repair pathway on cell survival, wild-type and *FANC*-deficient cells were exposed to IPMS, nPMS or MMS with and without pretreatment with O6-benzyl guanine (OBG), an inhibitor of MGMT. Pretreatment with OBG did not change the LC_50_ values of IPMS in wild-type or *FANC*-deficient cells, while OBG potentiated the cytotoxicity of nPMS and MMS in all of the cells examined. These results suggest that MGMT is not involved in the repair of O6-isopropyl dG and does not rescue cells from death, but instead repairs O6-n-propyl dG and O6-methyl dG and promotes cell survival. These findings are consistent with the report that O6-n-propyl dG is recognized by MGMT as efficiently as O6-methyl guanine and O6-ethyl dG [25]. Taken together, the sensitivity of *FANC*-deficient cells to IPMS, nPMS, MMS and EMS is associated with MGMT recognition activity of the adduct type that is generated.

Consistent with the observation that O6-isopropyl dG escapes recognition and repair by MGMT, IPMS-treated cells accumulated more DNA damage than nPMS-, MMS- or EMS-treated cells and thus demonstrate higher cytotoxicity. These unrepaired DNA lesions are assumed to lead to MN formation. Our PoD analysis in wild-type and *FANC*-deficient cells revealed that the FANC pathway suppresses IPMS-induced MN formation. In the DDR assay using DT40 mutant cells, *BRCA2*, *FANCD2*, *FANCI*, *FANCG*, *FANCL*, *REV1*, *REV3*, *RAD9*, *RAD18* and *PCNA* *K164R* mutants were hypersensitive to IPMS. Among these cells, the FANC pathway mutants were exclusively sensitive to IPMS while *REV1*, *REV3*, and *PCNA K164R* mutants were comparably sensitive to IPMS, MMS or EMS. These results suggest that the FANC repair pathway plays a pivotal role in repairing or tolerating IPMS-induced DNA damage compared to the other genes universally involved in repair or tolerance of alkylated DNA damages. Exogenous expression of the *FANCD2* gene in *FANCD2*-deficient cells restored the resistance to IPMS. The FANC pathway is known to function in DNA cross-link repair, homologous recombination and resolution of the replication arrest [29, 30]. If O6-methyl dG is left unrepaired, DNA replication over the O6MeG results in an O6MeG:T mismatch or O6MeG:C ambiguous pair [31]. Subsequently, the mismatch repair pathway works to fix the mismatch. However, since O6MeG remains in the template, a futile repair loop can eventually result in highly toxic DSBs during DNA replication [32, 33]. The same cellular response likely takes place in IPMS-treated cells. IPMS may generate mismatch repair pathway-mediated and replication-associated DSBs, which appear to be repaired by the FANC pathway. Monoubiquitylation of FANCD2 and FANCI is critical for downstream FANC pathway activation in the repair and tolerance of crosslinking DNA damage [34]. Monoubiquitination of FANCD2 and FANCI is catalyzed by the FANC core complex, including FANCG and FANCC, through the ubiquitin ligase subunit FANCL [35]. Our results show that DT40 cells deficient in *FANCD2*, *FANCI*, *FANCG*, or *FANCL* are hyper-sensitive to IPMS as compared to MMS and EMS, and monoubiquitination-deficient FANCD2 mutant cells are also markedly sensitive to IPMS. This suggests that mismatch repair pathway-coupled O6-alkylguanine-mediated DSBs during DNA replication activates the Fanconi anemia core complex and leads to the monoubiquitination of FANCD2 and FANCI. DT40 mutants deficient in *BRCA2* were also hyper-sensitive to IPMS, indicating that the monoubiquitination of FANCD2 and FANCI further stimulates downstream components of the FANC pathway to repair and tolerate DSBs caused by O6-alkylguanine. Interestingly, both *RAD18* and *RAD9*-deficient DT40 cells showed a similar trend of toxicity with lesser extent than *FANC*-deficient cells, suggesting that these DDR pathways could involve the upstream events of FANC pathway. Indeed, the FANC pathway is activated by *RAD18*, which also interacts with ubiquitinated chromatin components and facilitates *RAD9* recruitment to DNA DSBs [35, 36].

As unrepaired DSBs result in the formation of MN after cell division, we examined MN induction in cells treated with IPMS and nPMS in the presence and absence of OBG. The MN assay revealed that MGMT dysfunction elevated the MN induction frequency in wild-type cells treated with nPMS but not IPMS. MGMT did not affect MN frequency in *FANCD2*-deficient cells. These results indicate that O6-n-propyl dG accumulates in the absence of MGMT activity, but loss of MGMT activity does not change the number of O6-isopropyl dG adducts that remains on the DNA and eventually form MN or kill cells. An increase in MN frequency was not observed in OBG-pretreated *FANCD2* cells after treatment with nPMS. High spontaneous MN frequency in *FANCD2* cells, which is consistent with a previous report [37], may make it challenging to detect the small increases in MN frequency by MGMT inhibition. The increase in time-dependent accumulation of DSBs in *FANCD2*-deficient cells compared to wild-type cells may explain the stronger cytotoxicity and higher incidence of MN formation in *FANC*-deficient cells treated with IPMS. It is worthwhile to note that spontaneous MN frequency in *FANCG-*, *FANCI-*, and *FANCL-*deficient DT40 cells were 1.8, 3.4, and 6.6 times higher, respectively, than that in wild-type DT40 cells. Due to markedly high spontaneous MN frequency, the relative increase in MN frequency in *FANCL-*deficient DT40 cells is comparable to that in wild-type DT40 cells (Figure 5A). In contrast, the PoD concentration, which is indicative of a biologically significant increase, differed between wild type cells and *FANCL-*deficient cells. *FANCL-*deficient cells showed a lower value than that in wild type cells, which suggest *FANCL-*deficient cells are more sensitive to MN induction by IPMS.

The observation that the isopropyl moiety is a key structure that escapes recognition by MGMT can potentially be applied to cancer chemotherapy, since recent clinical trials have been undertaken to test DNA repair inhibitors that target PARP, BER, or MGMT in combination with alkylating agents [38]. In the case of OBG, a phase I clinical trial has defined the maximum tolerated dose of a single dose of TMZ when combined with OBG and has determined the dose of OBG that depletes tumor MGMT activity for 48 h [39]. Since IPMS causes MGMT-resistant O6-isopropyl dG and potential BER substrates such as N7-isopropyl dG and N3-isopropyl dA, co-treatment of IPMS and PARP inhibitor could cause synthetic lethality in HR-deficient tumor cells due to accumulation of DSBs caused by O6-isopropyl dG and induced by PARP1-DNA strand break complex during DNA replication. Therefore, incorporation of the isopropyl moiety may be worthwhile to test in future clinical trials, or test as a cancer chemotherapeutic approach, although the success of such approaches will of course depend on selective targeting of the tumor.

Overall, our findings in the present study reveal that IPMS-induced DNA damage escapes recognition by MGMT and generates DSBs. This leads to an activation of cell cycle checkpoints and results in FANC-mediated DSB repair.

## MATERIALS AND METHODS

### Chemicals

Isopropyl methanesulfonate (IPMS), ethyl methanesulfonate (EMS), methyl methanesulfonate (MMS), 2,3-bis (2-methoxy-4-nitro-5-sulfophenyl)-5-[(phenylamino) carbonyl]-2H-tetrazolium hydroxide (XTT), 1-methoxy-5-methylphenazinium methyl sulfate (PMS), O^6^-benzylguanine and dimethylsulfoxide (DMSO) were obtained from Sigma. Propyl methanesulfonate (nPMS) was purchased from Tokyo Chemical Industry Co., Ltd. (Tokyo, Japan). Dulbecco's phosphate buffered saline (PBS) was obtained from Life Technologies (Grand Island, NY, USA). All of the chemicals were dissolved in PBS, except XTT which was dissolved in DMSO.

### DT40 cell culturing and maintenance

Fetal bovine serum (FBS) and penicillin/streptomycin were obtained from Atlanta Biologicals (Norcross, GA, USA) and Sigma-Aldrich (St. Louis, MO, USA), respectively. RPMI 1640 culture medium (+glutamine, −phenol red) and chicken serum (CS) were acquired from Life Technologies (Grand Island, NY, USA). FBS and CS were heat inactivated at 56°C for 30 min. DT40 cells were maintained as described in our previous report [13]. The DT40 cell line and its isogenic mutants were knocked out in specific DNA repair pathways (Supplementary Table S1).

### Generation of FANCD2 knockout TK6 cell lines and cell cultivation

To disrupt the *FANCD2* gene, the guide RNA sequence (5′-TTTGGAAAGTGGTTGCTTCC-3′) was designed against exon 2 and cloned into the pX330 (Addgene) plasmid after digestion with *Bbs*I. To generate the targeting vectors, the left and right arms were amplified by polymerase chain reaction (PCR) using 5′-GCGAATTGGGTACCGGGCCCATTGAGTCTCAAATTTGGT-3′/5′-CTGGGCTCGAGGGGGGGCCAATATGGCAAATAGTAAAGG-3′ (for the left arm), and 5′-TGGGAAGCTTGTCGACTTAACAGAATCAACTAGGTAATAT-3′/5′-ACTAGTAGGCGCGCCTTAAGCTTCTTTTGGAAAGCTATT-3′ (for the right arm), respectively. The amplified fragments of the left and right arms were assembled by Seamless Cloning (A14606, Invitrogen) into the *DT-ApA*/*NEO^R^* (provided from the Laboratory for Animal Resources and Genetic Engineering, Center for Developmental Biology, RIKEN Kobe, http://www.cdb.riken.jp/arg/cassette.html) and *DT-ApA*/*PURO^R^* cassettes [40] which were digested with *ApaI* and *AflII*. The single underlines seen above indicate the homology of upstream and downstream from *ApaI* site. The double underlines above indicate the homology upstream and downstream from *AflII* site. The gene disruption was confirmed via immunoblot using an anti-FANCD2 antibody (sc-2022, Santa Cruz). β-actin (A5316, Sigma) was used as a loading control. TK6 cells were maintained in RPMI 1640 culture medium (+glutamine, −phenol red) supplemented with FBS (10%) and penicillin/streptomycin.

### DT40 cell-based DNA damage response (DDR) assay

For the DDR assay, approximately 1,200 cells were seeded in each well of a 96-well plate in 75 μL of medium. The wild-type and mutant cells were seeded in one plate, and the cells were treated with IPMS, MMS or EMS in duplicate wells for each chemical. The outside 2 wells were used as blank controls. These plates were kept in an incubator at 39.5°C until the start of treatment. Separately, each chemical was serially diluted into eight concentrations. 8.35 μL of the chemical solutions or PBS (as a solvent control, 2 wells/row) were added to 2 rows of a 96-well plate. A preliminary dose finding test was conducted in DT40 wild type cells to select the appropriate concentration range, which included at least one concentration showing severe cytotoxicity and one concentration showing no cytotoxicity at a lower concentration.

After the chemical solutions were added, plates were incubated for at least 48 hours after which they were intermittently observed microscopically to determine the growth of the cells. Once the control cells covered 1/3 or more of a well bottom, cells were exposed to the XTT cocktail. The plates were returned to the incubator until the dye developed sufficient color for detecting absorbance on the plate reader, which normally takes 2-5 hours. Absorbance was measured at 450 nm with a reference of 620 nm using a Tecan Sunrise (Tecan Systems, San Jose, CA, USA) plate reader with XFluor^™^ software (Tecan, version 6.4). All data are summarized as the means of experiments done at least in triplicate.

### Micronucleus assay

For the micronucleus assay, flow cytometry-based MN measurements were performed using an *In Vitro* MicroFlow™ Kit (Litron Laboratories, Rochester, NY, USA) according to the manufacturer's instructions [41]. Briefly, 150 μL of DT40 cells adjusted to 2 × 10^5^ cells/ml were seeded into 96-well plates at 39°C and 5% CO_2_ in a humid atmosphere. Chemical solutions were separately prepared, and 16.7 μL of the solutions were added to each well. Cells were cultured at 39°C for 16 hours, which corresponds to approximately two cell cycles. After treatment, the plate was centrifuged to collect cells, and the treatment medium was discarded. The plate was then placed on ice and incubated for 20 min, and 50 μL of Nucleic Acid DyeA working solution was added to each well. A light source was placed approximately 15 cm above the samples for 30 min. Subsequently, the samples were washed once with cold 1x Buffer Solution. Next, 100 μL of Complete Lysis Solution 1 (including Nucleic Acid DyeB) were added to each well, and the samples were incubated for 1 hour in the dark at 37°C. Subsequently, 100 μL of Complete Lysis Solution 2 (including Nucleic Acid DyeB) were added to each well, and the samples were incubated for 30 min in the dark at room temperature.

Samples for the MN assay were analyzed by flow cytometry (FACS Calibur or LSRII, BD Biosciences) using an excitation wavelength of 488 nm. The frequency of MN was determined by acquiring at least 10,000 gated nuclei per sample. The data is summarized as a mean of two or more independent experiments. The extent of survival in the treated cultures was evaluated as the reduction in population doubling (PD). This is described as relative population doubling (RPD), which is calculated using the following formula:

(Number of PD in treated cultures)/(Number of PD in control cultures) × 100,

where PD is calculated as

[log(post-treatment cell number/initial cell number)]/log 2.

### Pretreatment with O^6^-benzylguanine

Cells were pretreated with O^6^-benzylguanine (OBG) and used for both the DDR and MN assays to see if MGMT inhibition changes the cellular response. Prior to treatment, cells were pre-incubated with 10 μM of OBG for 2 hours and exposed to chemicals. No additional OBG was supplemented during the exposure.

### γH2AX assay

To determine the percentage of γH2AX-positive cells, IPMS-treated cells were harvested and stained with a FITC conjugated γH2AX antibody using an H2A.X phosphorylation Assay Kit for Flow Cytometry (Millipore Corporation, CA, USA) according to the manufacturer's instructions. Briefly, 1 × 10^6^ cells were exposed to chemicals and harvested after a 1, 2, 4, 8 or 16 hour treatment period. Cells were washed once with PBS and resuspended in 200 μL of 1X Fixation solution and stored on ice for 20 minutes. After fixation, cells were washed with PBS and resuspended in 100 μL of 1X Permeabilization solution, in which 3.5 μL of anti-phospho-Histone H2A.X (Ser139) was added. After incubation on ice for 20 minutes, 100 μL of 1X Wash solution was added. The cells were harvested by centrifugation and resuspended in 200 μL of PBS. The samples were analyzed by flow cytometry (FACS Calibur, BD Biosciences), and the data was analyzed by FlowJo (Ver. X, Tree Star, Inc.).

### Immunofluorescence

TK6 cells (0.5 million/ml) were exposed to IPMS (750μM) for 24 hours. Cells were then fixed using 2% formaldehyde / PBS for 20 min, washed in PBS, and spotted on to double frosted microscopic glass slides (Fisher Scientific) at a concentration of 2 million/ml. Blocking was achieved using 5% (w/v) bovine serum albumin (BSA) in PBS-TT for 30 min. Cells were then incubated with 1:500 rabbit polyclonal anti-53BP1 antibody (Bethyl Laboratories, Montgomery, TX) and mouse γH2AX antibody (Millipore Corporation, CA, USA) in 1% BSA/PBS-TT for overnight at 4^o^C. Cells were then washed in PBS, incubated in 1:250 anti-mouse Alexa Fluor 555 (Life Technologies) and Alexa Fluor 488 antibodies (Jackson ImmunoResearch Laboratories, West Grove, PA) for 1h. After washing with PBS, slides were mounted with a cover slip using Vectashield with DAPI (Vector Laboratories) and sealed using nail polish. Slides were analyzed using confocal microscopy (Zeiss CLSM 700). Optical sections through the nuclei were captured at 0.5 μm intervals, and the images were obtained by maximum projection of the individual sections.

### Data and statistical analysis

For DDR assay data sets, we calculated the lethal concentration 50 (LC_50_) values for each cell line using Graphpad Prism 5 (La Jolla, CA, USA). The obtained LC_50_ in each mutant was compared against the LC_50_ in wild-type cells.

In order to represent the point of departure values for concentration-response in the MN assay in wild-type and *FANC*-deficient cells, the benchmark dose (BMD) values (U.S.EPA/100/R-12/001 2012) were obtained using the EPA BMD software v 2.5. These values represent a one standard deviation departure from control values, and the BMDL refers to the corresponding lower limit of a one-sided 95% confidence interval on the BMD. A log_10_ (concentration+0.5) was used to avoid taking logarithms of zero control values. In each instance, the best fitting result from Hill, exponential, and polynominal response non-constant variance models was used.

Fisher's exact test was used to examine whether the micronucleus frequency differed between the OBG pretreated cells and untreated cells following IPMS or nPMS treatment. The results were determined to be statistically significant when the *P*-value was less than 0.05.

## SUPPLEMENTARY FIGURE AND TABLE




