# Supplementary material for: Poor recognition of O6-isopropyl dG by MGMT triggers double strand break-mediated cell death and micronucleus induction in FANC-deficient cells
Source: Oncotarget. 2016 Jul 29;7(37):59795–808. doi: 10.18632/oncotarget.10928 (PMC5312349; doi:10.18632/oncotarget.10928)
Supplement: Supplementary file 1 [file oncotarget-07-59795-s001.pdf]

Poor recognition of O6-isopropyl dG by MGMT triggers double strand break-mediated cell death and micronucleus induction in *FANC*-deficient cells

SUPPLEMENTARY FIGURE AND TABLE

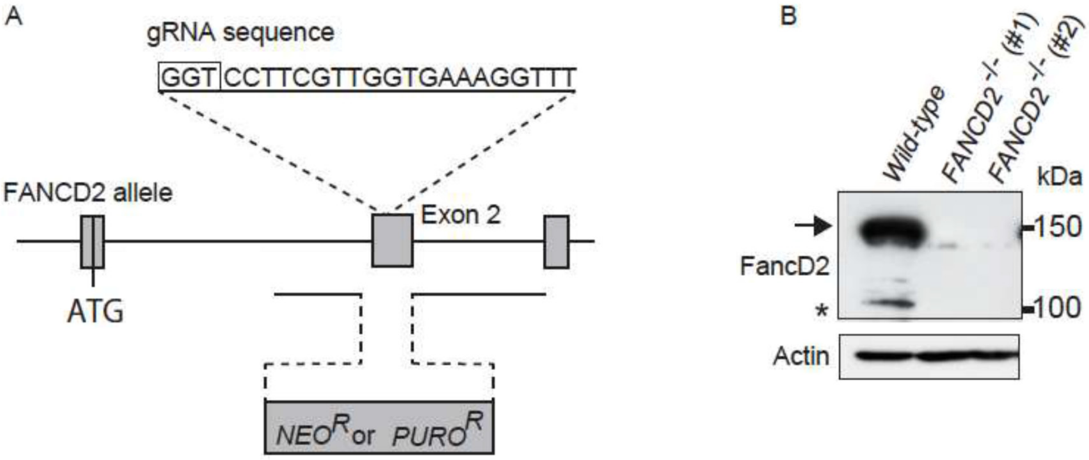

Supplementary Figure S1: Generation of FANCD2 knockout TK6 cell line. A. Schematic of *FANCD2*-gene-disruption and the sequence of guide RNA(gRNA). Black frame in gRNA sequence indicates PAM. Exon2 was targeted and replaced with the targeting vectors having antibiotics makers. **B.** Western blot analysis of Fancd2 in TK6 whole cell extract using FANCD2 antibody. The arrow and asterisk indicate the predicted molecular size of Fancd2 and the degradation protein of Fancd2, respectively. β-actin is a loading control.

Supplementary Table S1: DT40 mutant cells used in this study.

See Supplementary File 1
