# Supplementary material for: Poor recognition of O6-isopropyl dG by MGMT triggers double strand break-mediated cell death and micronucleus induction in FANC-deficient cells
Source: Oncotarget. 2016 Jul 29;7(37):59795–808. doi: 10.18632/oncotarget.10928 (PMC5312349; doi:10.18632/oncotarget.10928)
Supplement: Supplementary file 2 [file oncotarget-07-59795-s002.docx]

**Table S1.** DT40 mutant cells used in this study.

| Gene | Function | References |
| --- | --- | --- |
| POLB | Base excision repair (BER) | (1) |
| FEN1 | BER, processing of 5' flap during DNA replication | (2) |
| PARP1 | Poly(ADP-ribosyl)ation, BER, single strand break (SSB) and ouble strand break (DSB) repair | (3) |
| BLM | RecQ Helicase | (18) |
| WRN | RecQ Helicase | (19) |
| XPA | Nucleotide excision repair (NER) | (4) |
| XPG | NER, transcription-coupled BER | (20) |
| KU70 | Non-homologous end joining (NHEJ) | (21) |
| LIGIV | NHEJ | (11) |
| REV1 | Translesion DNA synthesis (TLS) | (22) |
| REV3 | TLS | (14) |
| RAD18 | Regulation of TLS, ubiquitin E3 ligase | (17) |
| POLH | TLS | (15) |
| POLK | TLS | (8) |
| POLQ | TLS, Base excision repair | (16) |
| RAD54 | Homologous recombination (HR) | (10) |
| BRCA1 | HR | (23) |
| BRCA2 | HR | (9) |
| FANCC | Damage response to interstrand cross-links | (25) |
| FANCD2 | Damage response to interstrand cross-links | (5) |
| FANCI | Damage response to interstrand cross-links | (6) |
| FANCG | Damage response to interstrand cross-links | (7) |
| FANCL | Damage response to interstrand cross-links | (8) |
| ATM | Cell-cycle checkpoint control as sensors | (13) |
| RAD9 | Cell-cycle checkpoint control as sensors | (12) |
| CTIP | DSB repair, DNA resection | (24) |
| PCNA-K164R | PCNA mutant deficient in ubiquitination at 164 lysine: TLS | (26) |
| TDP1 | Tyrosyl-DNA phosphodiesterase 1: repairing stalled topoisomerase I-DNA complex | (27) |
| FANCD2+  Wt FANCD2 | FancD2 mutant cells expressing wild-type FancD2 | (28) |
| FANCD2-KR | FancD2 carrying a monoubiquitination site mutation (K563R) | (28) |

**SUPPLEMENTAL REFERENCES:**

1. Tano, K., Nakamura, J., Asagoshi, K., Arakawa, H., Sonoda, E., Braithwaite, E. K., Prasad, R., Buerstedde, J. M., Takeda, S., Watanabe, M. and Wilson S. H. (2007) Interplay between DNA polymerases beta and lambda in repair of oxidation DNA damage in chicken DT40 cells. *DNA Repair (Amst)*, **6**, 869-875.

2. Matsuzaki, Y., Adachi, N. and Koyama, H. (2002) Vertebrate cells lacking FEN-1 endonuclease are viable but hypersensitive to methylating agents and H2O2. *Nucleic Acids Res*, **30**, 3273-3277.

3. Hochegger, H., Dejsuphong, D., Fukushima, T., Morrison, C., Sonoda, E., Schreiber, V., Zhao, G. Y., Saberi, A., Masutani, M., Adachi, N. *et al.* (2006) Parp-1 protects homologous recombination from interference by Ku and Ligase IV in vertebrate cells. *EMBO J.*, **25**, 1305-1314.

4. Okada, T., Sonoda, E., Yamashita, Y. M., Koyoshi, S., Tateishi, S., Yamaizumi, M., Takata, M., Ogawa, O. and Takeda, S. (2002) Involvement of vertebrate polkappa in Rad18-independent postreplication repair of UV damage. *J. Biol. Chem.*, **277**, 48690-48695.

5. Yamamoto, K., Hirano, S., Ishiai, M., Morishima, K., Kitao, H., Namikoshi, K., Kimura, M., Matsushita, N., Arakawa, H., Buerstedde, J. M. *et al.* (2005) Fanconi anemia protein FANCD2 promotes immunoglobulin gene conversion and DNA repair through a mechanism related to homologous recombination. *Mol. Cell Biol.*, **25**, 34-43.

6. Ishiai, M., Kitao, H., Smogorzewska, A., Tomida, J., Kinomura, A., Uchida, E., Saberi, A., Kinoshita, E., Kinoshita-Kikuta, E., Koike, T., Tashiro, S., Elledge, S. J. and Takata, M. (2008) FANCI phosphorylation functions as a molecular switch to turn on the Fanconi anemia pathway. *Nat. Struct. Mol. Biol.*, **15**, 1138-1146.

7. Yamamoto, K., Ishiai, M., Matsushita, N., Arakawa, H., Lamerdin, J. E., Buerstedde, J. M., Tanimoto, M., Harada, M., Thompson, L. H. and Takata, M. (2003) Fanconi anemia FANCG protein in mitigating radiation- and enzyme-induced DNA double-strand breaks by homologous recombination in vertebrate cells. *Mol. Cell Biol.*, **23**, 5421-5430.

8. Matsushita, N., Kitao, H., Ishiai, M., Nagashima, N., Hirano, S., Okawa, K., Ohta, T., Yu, D. S., McHugh, P. J., Hickson, I. D., Venkitaraman, A. R., Kurumizaka, H. and Takata, M. (2005) A FancD2-monoubiquitin fusion reveals hidden functions of Fanconi anemia core complex in DNA repair. *Mol. Cell*, **19**, 841-847.

9. Hatanaka, A., Yamazoe, M., Sale, J. E., Takata, M., Yamamoto, K., Kitao, H., Sonoda, E., Kikuchi, K., Yonetani, Y. and Takeda, S. (2005) Similar effects of Brca2 truncation and Rad51 paralog deficiency on immunoglobulin V gene diversification in DT40 cells support an early role for Rad51 paralogs in homologous recombination. *Mol. Cell Biol.*, **25**, 1124-1134.

10. Bezzubova, O., Silbergleit, A., Yamaguchi-Iwai, Y., Takeda, S. and Buerstedde, J. M. (1997) Reduced X-ray resistance and homologous recombination frequencies in a RAD54-/- mutant of the chicken DT40 cell line. *Cell*, **89**, 185-193.

11. Adachi, N., Ishino, T., Ishii, Y., Takeda, S. and Koyama, H. (2001) DNA ligase IV-deficient cells are more resistant to ionizing radiation in the absence of Ku70: Implications for DNA double-strand break repair. *Proc. Natl. Acad. Sci. U. S. A.*, **98**, 12109-12113.

12. Kobayashi, M., Hirano, A., Kumano, T., Xiang, S.L., Mihara, K., Haseda, Y., Matsui, O., Shimizu, H. and Yamamoto, K. (2004) Critical role for chicken Rad17 and Rad9 in the cellular response to DNA damage and stalled DNA replication. *Genes Cells*, **9**, 291-303.

13. Takao, N., Kato, H., Mori, R., Morrison, C., Sonada, E., Sun, X., Shimizu, H., Yoshioka, K., Takeda, S and Yamamoto, K. (1999) Disruption of ATM in p53-null cells causes multiple functional abnormalities in cellular response to ionizing radiation. *Oncogene*, **18**, 7002-7009.

14. Sonoda, E., Okada, T., Zhao, G. Y., Tateishi, S., Araki, K., Yamaizumi, M., Yagi, T., Verkaik, N.S., van Gent, D. C., Takata, M. and Takeda S. (2003) Multiple roles of Rev3, the catalytic subunit of polzeta in maintaining genome stability in vertebrates. *EMBO J.*, **22**, 3188-3197.

15. Kawamoto, T., Araki, K., Sonoda, E., Yamashita, Y. M., Harada, K., Kikuchi, K., Masutani, C., Hanaoka, F., Nozaki, K., Hashimoto, N. and Takeda S. (2005) Dual roles for DNA polymerase eta in homologous DNA recombination and translesion DNA synthesis. *Mol. Cell.*, **20**, 793-799.

16. Yoshimura, M., Kohzaki, M., Nakamura, J., Asagoshi, K., Sonoda, E., Hou, E., Prasad, R., Wilson, S. H., Tano, K., Yasui, A. *et al.* (2006) Vertebrate POLQ and POLbeta cooperate in base excision repair of oxidative DNA damage. *Mol. Cell*, **24**, 115-125.

17. Yamashita, Y.M., Okada, T., Matsusaka, T., Sonoda, E., Zhao, G.Y., Araki, K., Tateishi, S., Yamaizumi, M. and Takeda, S. (2002) RAD18 and RAD54 cooperatively contribute to maintenance of genomic stability in vertebrate cells. *EMBO J*, **21**, 5558-5566.

18. Wang W, Seki M, Narita Y, Sonoda E, Takeda S, Yamada K, Masuko T, Katada T, Enomoto T. (2000) Possible association of BLM in decreasing DNA double strand breaks during DNA replication. EMBO J 19:3428–435.

19. Imamura O, Fujita K, Itoh C, Takeda S, Furuichi Y, Matsumoto T. (2002) Werner and Bloom helicases are involved in DNA repair in a complementary fashion. Oncogene 21:954–963.

20. Kikuchi K, Taniguchi Y, Hatanaka A, Sonoda E, Hochegger H, Adachi N, et al. (2005) Fen-1 facilitates homologous recombination by removing divergent sequences at DNA break ends. Mol Cell Biol 25(16):6948–6955.

21. Takata M, Sasaki MS, Sonoda E, Morrison C, Hashimoto M, Utsumi H, et al. (1998) Homologous recombination and non-homologous endjoining pathways of DNA double-strand break repair have overlapping roles in the maintenance of chromosomal integrity in vertebrate cells. EMBO J 17:5497–5508.

22. Simpson LJ, Sale JE. (2003) Rev1 is essential for DNA damage tolerance and non-templated immunoglobulin gene mutation in a vertebrate cell line. EMBO J. 22:1654-1664.

23. Martin RW, Orelli BJ, Yamazoe M, Minn AJ, Takeda S, Bishop DK. (2007) RAD51 up-regulation bypasses BRCA1 function and is a common feature of BRCA1-deficient breast tumors. Cancer Res. 67:9658-9665.

24. Nakamura K, Kogame T, Oshiumi H, Shinohara A, Sumitomo Y, Agama K, Pommier Y, Tsutsui KM, Tsutsui K, Hartsuiker E, Ogi T, Takeda S, Taniguchi Y. (2010) Collaborative action of Brca1 and CtIP in elimination of covalent modifications from double-strand breaks to facilitate subsequent break repair. PLoS Genet. ;6:e1000828

25. Hirano S, Yamamoto K, Ishiai M, Yamazoe M, Seki M, Matsushita N, Ohzeki M, Yamashita YM, Arakawa H, Buerstedde JM, Enomoto T, Takeda S, Thompson LH, Takata M. (2005) Functional relationships of FANCC to homologous recombination, translesion synthesis, and BLM. EMBO J. 24:418-27.

26. Arakawa H, Moldovan GL, Saribasak H, Saribasak NN, Jentsch S, Buerstedde JM. (2006) A role for PCNA ubiquitination in immunoglobulin hypermutation. PLoS Biol. 4:e366.

27. Murai J, Huang SY, Das BB, Dexheimer TS, Takeda S, Pommier Y. Tyrosyl-DNA phosphodiesterase 1 (TDP1) repairs DNA damage induced by topoisomerases I and II and base alkylation in vertebrate cells. (2012) J Biol Chem. 287:12848-57.

28. Matsushita N, Kitao H, Ishiai M, Nagashima N, Hirano S, Okawa K, Ohta T, Yu DS, McHugh PJ, Hickson ID, Venkitaraman AR, Kurumizaka H, Takata M. (2005) A FancD2-monoubiquitin fusion reveals hidden functions of Fanconi anemia core complex in DNA repair. Mol Cell. 5;19:841-7.
